# The trajectory for software-based medical devices

**DOI:** 10.3389/fmedt.2023.1195929

**Published:** 2023-04-17

**Authors:** Dinesh Kumar

**Affiliations:** School of Engineering, RMIT University, Melbourne, VIC, Australia

**Keywords:** software based medical devices, medical robot, medical apps, future of health care, AI in medicine

The list of new inventions in the field of medical devices appears to be endless. The growth in the field has resulted in seemingly limitless potential. Now, more than ever before it is possible to imagine a world where the quality of healthcare is not based on the size of one's income, or the post-code where the individual resides. The large differences in the health based on race and ethnicity between urban and rural areas have the potential to disappear.

While there is large diversity in medical devices and their development is endless, the rapid progress in three fields that stand out are: Medical robotics, AI based medical devices and Software As a Medical Device (SAMD). Whether it is AI based software or smartphone apps, these devices have one common theme—they are Software driven. Often, these use commercially available hardware, and the uniqueness is the software. These are collectively known as Software Based Medical Devices (SBMD).

SBMDs have impacted all aspects of the lives of millions of people. An amputee with no hands can have a functioning hand, thanks to the smart prosthetic hand. Smart wheelchairs can assist people with brain injuries to live with minimal assistance, robotic nurses can support the elderly living with dignity, and SBMDs reduce multi-day surgeries into mere hours. An orthopaedic surgeon can print a hip implant to the exact needs of a patient while smartphones can monitor the efficacy of the medicine dose for the person with Parkinson's disease. And a capsule carries wireless sensors and provides the details of the digestive tracks of the person. Clinicians can treat each patient as an individual, and monitor their progress exactly.

Progress in SBMD for medical imaging has been in the development of imaging techniques, their analysis and transmission. Complex diagnoses are now performed without harmful radiation. MRIs can detect diseases that were undetectable until recently, and microelectronics have provided implants that can continuously monitor brain activity. An OCT can view the retina of the eyes, while an ultrasound can view the foetus for any abnormality.

Medical Robotics, referred to as Medrobotics, is another area of medical breakthrough. Ranging from the exoskeletal devices for post-stroke to highly advanced robotic devices that can be used for performing complex surgical procedures. Procedures that used to take hours and require weeks of post-operative phase have now become single day surgeries. Miniaturised medical robotics can be implanted for procedures such as exact medication.

AI has been embedded in medical devices for over 4 decades. Recent advancement has progressed these devices and supports personalised healthcare which treats people as individuals. SBMDs not only provide the individual with personalised lifestyle and diet management but also with monitoring and monitoring of symptoms and medicine dosage. Personalized medicine has finally arrived.

One of the big advantages of SBMDs is the ease at which they can be developed. There are a large number of platforms that support the development of smartphone based apps and websites that can be easily launched to reach the target users. With the development and easy to access AI tools such as by Google and Microsoft, developing a model does not appear to be a very difficult task. Easy to access cameras, mechanical and chemical sensors, and devices such as 3D printers, the use of SBMD for medical applications is only limited by your imagination.

However, with great opportunities come equal concerns. The ease with which AI models can be developed suggests that such models can be developed without proper understanding and such models may be unsuitable. The tools that allow easy development of medical apps can result in unsuitable SAMD. There are platforms that allow the development and marketing of such Apps without oversight of the clinical validity, and thus while some of these are useful, many are useless but benign, but there are also some that are truly harmful.

There are also other issues of concern such as who owns the Intellectual Property (IP)? What are the issues of bias in the models? How to protect the IP? Then the concerns regarding transparency of the potential data bias. Who is responsible for the training of the clinicians in use of SBMD? How to ensure the developers are compensated for their efforts and held to account if things go badly? Government bodies such as TGA in Australia, and FDA in the USA are developing guidelines for managing these devices. However, the challenge for the local authorities can be that many of these are available online and thus difficult to police.

SBMDs have the potential of revolutionising the medical technological landscape transforming risk assessments of disease conditions, disease diagnosis, disease monitoring, treatment and management. They can provide personalised care and reduce healthcare disparities for urban and rural people, and for people from richer and less affluent societies. However, with this growth are also the challenges such as data management and security, ethics of data ownership, the legality of error, transparency with a clinician and clinical training. Thus, there is a need for scrutiny of these devices, and the only real option for this is by using the yardstick of high-quality peer review process. It is thus essential to attract work in this area to our journals.

There is also the need to attract transformative talent to realise innovative solutions to overcome the current limitations in SBMD. If we do not address these challenges soon, the space will be crowded by Cowboys, and SBMD will lose credibility. Therefore, we must develop enforceable guidelines to protect the public, scientists, and developers. This is where peer-reviewed journals can play a major role.

I believe that now is the time to discuss the topics of SBMD in scientific forums. Not only is this an opportunity to showcase our work, but we can also highlight the clinical validity of the work, and demonstrate the efficacy of these devices. This will lead to significant impact on the health and wellbeing of people, irrespective of their social status and distance from major urban centres.

